# Analysis of Bacterial Wilt Management Strategies From the Dynamic Perspective of Environmental Adaptation Approaches of *Ralstonia solanacearum*

**DOI:** 10.1111/1758-2229.70335

**Published:** 2026-04-03

**Authors:** Mingzhao Han, Xin Liu, Guixiang Li, Peng Li

**Affiliations:** ^1^ Ministry of Education Key Laboratory for Ecology of Tropical Islands, Key Laboratory of Tropical Animal and Plant Ecology of Hainan Province, College of Life Sciences Hainan Normal University Haikou China

**Keywords:** control strategies, environmental adaptation, integrated disease management, pathogenicity mechanisms, quorum sensing, resistance breeding

## Abstract

The *Ralstonia solanacearum* species complex (RSSC) ranks among the most destructive plant pathogens worldwide, due to its broad host range, extensive geographic distribution and remarkable environmental adaptability. Its persistence in soil and colonization of plant vascular tissues severely limits the effectiveness of conventional chemical control, posing significant challenges for disease management. This review highlights recent advances in understanding the environmental adaptation mechanisms of RSSC. Key topics include the dynamic evolution of pathogenicity, niche‐specific survival strategies and virulence regulation mediated by quorum sensing, and complex interactions with surrounding microbial communities that shape its behaviour and fitness. We further provide a comprehensive assessment of current control strategies from an ecological perspective, encompassing physical, chemical, genetic, agronomic and microbial approaches, with critical evaluation of their mechanisms, potential and limitations. Meanwhile, we discuss the major challenges in bacterial wilt management and outline future directions, with an emphasis on multi‐omics‐informed precision breeding, microbiome engineering and intelligent integrated disease management (IDM). These emerging strategies hold promise for the sustainable and effective long‐term control of bacterial wilt disease caused by RSSC.

## Graphical abstract

This review reframes bacterial wilt management by analysing control strategies through the lens of the pathogen's evolutionary, physiological and ecological adaptations. This mechanism‐based approach links pathogen traits to control efficacy, guiding the development of more durable solutions.

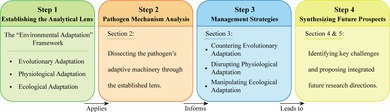

## 1 Introduction

Plant diseases pose a significant challenge to the sustainable development of global agriculture, with vascular diseases caused by soil‐borne pathogens being particularly problematic due to their concealed nature, persistence and severe consequences (Ristaino et al. 2021). The *Ralstonia solanacearum* species complex (RSSC) serves as a typical example and is known as the second detrimental bacterial plant pathogen worldwide (Mansfield et al. 2012), with an extensive host range, which can infect over 450 plant species spanning more than 50 families and yield great losses, including economically vital crops like tomato, potato, tobacco, banana, peanut and ginger (Vailleau and Genin 2023), thereby posing a substantial threat to both global food security and agricultural economies (Wang et al. 2023).

The *R. solanacearum* is a Gram‐negative β‐proteobacterium that typically enters host plants through natural root openings or wounds, subsequently colonizing the root cortex and invading the xylem vessels (Lowe‐Power et al. 2018). Within the vascular tissues, the bacterium multiplies rapidly, reaching extremely high densities (up to 10^9^–10^10^ CFU/g stem) and produces abundant extracellular polysaccharides (EPS). The accumulation of EPS physically occludes the vessels, thereby disrupting water and nutrient transport which results in wilting and often plant death (Yang et al. 2021; Prakasha et al. 2017). Notably, *R. solanacearum* can persist for extend periods in soil, water and weed hosts, severing as a continuous inoculum reservoir and posing severe challenge for disease management and eradication (Alvarez et al. 2008).

The RSSC exhibits remarkable genetic diversity and is currently classified into three species, which are closely associated with geographic origin and further subdivided into numerous sequevars (Sharma et al. 2022; Prior et al. 2016). This extensive genetic variability underlies its broad host range and poses significant challenges for resistance breeding and disease management. Conventional chemical treatments, including antibiotics and copper‐based compounds, often fail due to the pathogen's deep colonization in plant tissue and soil, and their overuse may contribute to resistance development and environmental contamination (Nion and Toyota 2015). As a result, the development of environmentally sustainable, cost‐effective and durable integrated management strategies has become a pressing priority in plant pathology and global agriculture. Recent advances in genomics, transcriptomics, metabolomics and other high‐throughput approaches have substantially enriched our understanding of *R. solanacearum* 's pathogenic mechanisms and adaptive strategies (Zeiss et al. 2019; Singh et al. 2025; Shi et al. 2022). This pathogen functions not only as a destructive invader but also as a highly adaptive “ecological strategist”. Through complex regulatory systems such as quorum sensing (QS), it precisely tunes its responses to environmental changes, orchestrating growth, reproduction and virulence (Kai 2023; Yan et al. 2022). Additionally, it actively manipulates the host microenvironment and engages in intricate interactions with the surrounding microbiota to enhance survival and persistence. Deciphering these multifaceted adaptive mechanisms is essential for elucidating its pathogenicity and developing effective, long‐term control measures.

Despite several recent reviews that have comprehensively summarized the pathogenic mechanisms and management strategies of *R. solanacearum* (Wang et al. 2023; Jiang et al. 2025; Abu Bakar et al. 2025), these discussions often consider pathogen biology and disease control as largely separate domains. A coherent framework that explicitly links the pathogen's core survival principles, particularly its exceptional environmental adaptability, to the performance of existing management strategies remains limited. Here, we synthesize current knowledge by viewing *R. solanacearum* as an ecological strategist and by examining disease management through the lens of pathogen adaptation. We propose that the effectiveness of diverse control strategies can be understood in terms of how they constrain, redirect, or exploit specific adaptive mechanisms. To systematically develop this perspective, we deconstruct the environmental adaptability of *R. solanacearum* into three interrelated dimensions. First, evolutionary adaptation enables the pathogen to continuously renew its virulence repertoire through horizontal gene transfer and genome remodelling, facilitating rapid responses to host immunity and environmental pressures. Second, physiological adaptation allows flexible switching between growth and virulence programs across ecological niches via sophisticated regulatory networks, such as quorum sensing, thereby optimizing resource allocation. Third, ecological adaptation operates at the community level, where the pathogen maximizes survival and infection success through competitive, cooperative and symbiotic interactions within complex microbial assemblages.

**FIGURE 1:**
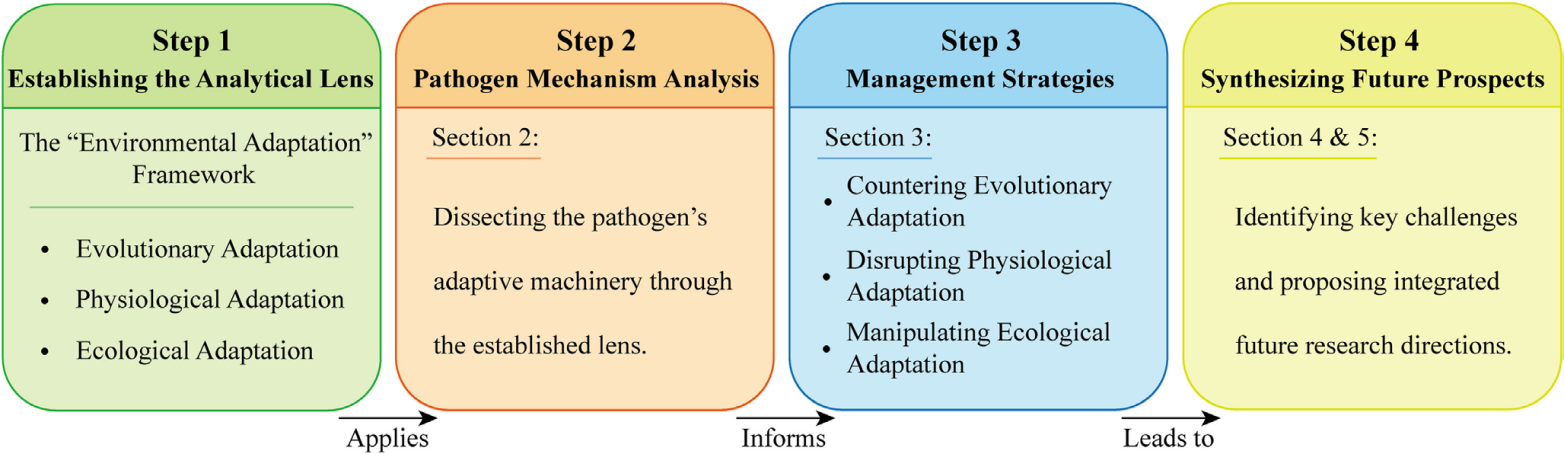
Conceptual roadmap illustrating the logical progression of the review.

Building upon this three‐dimensional framework, the present review is structured as follows (Figure 1). First, we will systematically dissect the key mechanisms underpinning *R. solanacearum's* evolutionary, physiological and ecological adaptation, paying special attention to the ecological trade‐offs that constrain its behaviour. Next, using this adaptation framework as an analytical lens, we will re‐evaluate a comprehensive suite of management strategies, shifting the focus from simple description to an analysis of their underlying ecological and mechanistic rationales. Specifically, we will demonstrate how each strategy succeeds or fails based on its ability to target a specific adaptive trait of the pathogen. Finally, by synthesizing these insights, we will identify critical challenges and propose future research directions aimed at developing more durable and ecologically‐informed management solutions.

## 2 Environmental Adaptation Mechanisms of RSSC

**FIGURE 2:**
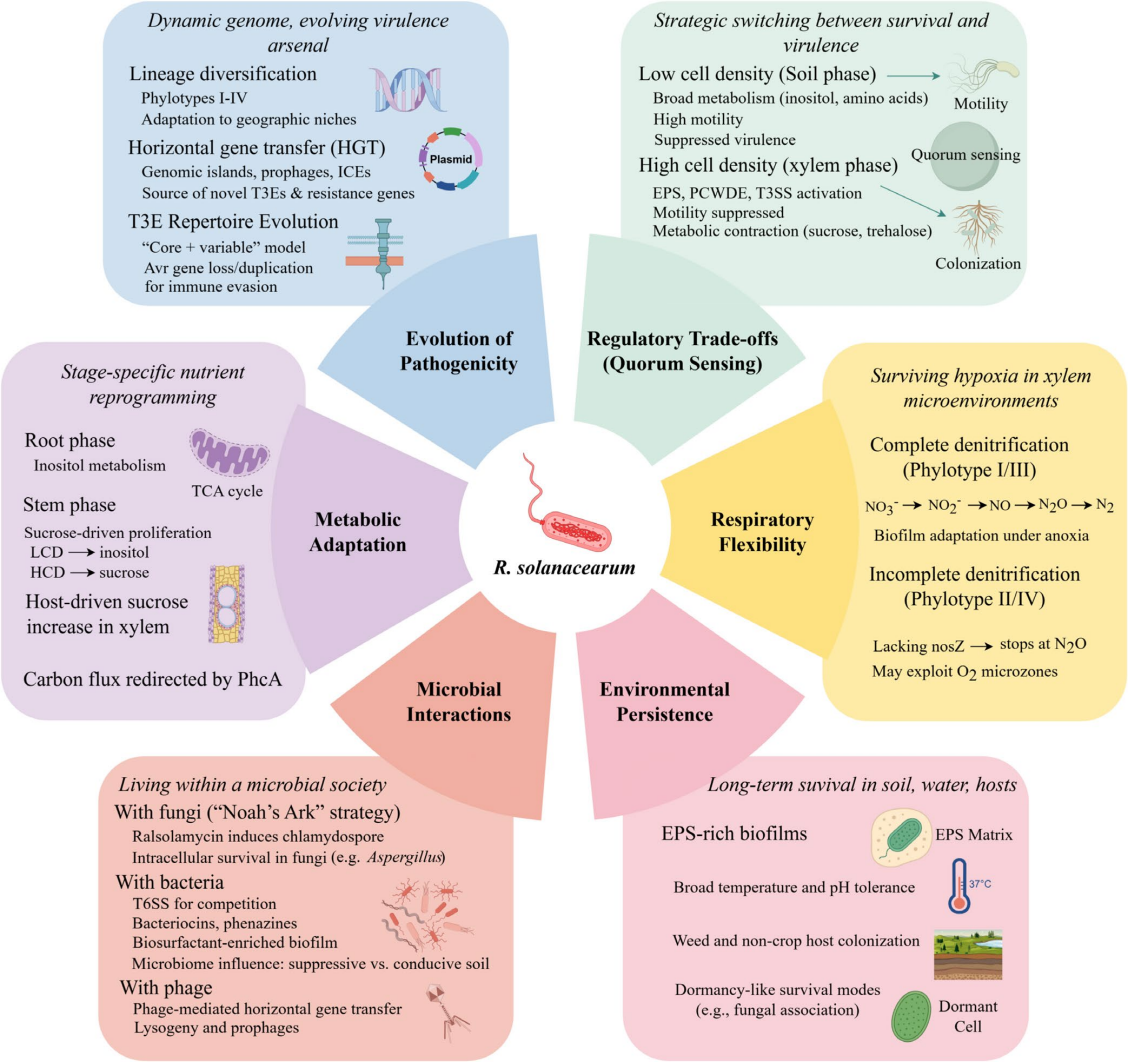
Environmental adaptation mechanisms of *R. solanacearum* .

The global success of *R. solanacearum* as a plant pathogen is largely attributed to its exceptional environmental adaptability. The *R. solanacearum* exhibits rapidly evolutionary capacity, ranging from macro‐scale evolutionary dynamics to micro‐scale molecular regulatory networks, which enable it to colonize diverse hosts and thrive in a wide range of environmental conditions (Figure 2). Moreover, under resource‐limited or stressful scenarios, the pathogen orchestrates finely tuned decisions between survival and virulence through complex regulatory circuits, while simultaneously engaging in multifaceted interactions with coexisting microbial communities.

### 2.1 Evolution of Pathogenicity: A Dynamic Arsenal

The pathogenicity of *R. solanacearum* is not a static attribute, but rather a dynamic trait continuously shaped by long‐term interaction with host plants and environmental pressures. Its genome functions as a flexible ‘arsenal’, subject to frequent updates and structural rearrangements through various mechanisms such as horizontal gene transfer, genomic island acquisition and recombination events.

#### 2.1.1 Species Complex Formation and Lineage Diversification

The phylogenetic diversification of the RSSC is closely linked to its geographic origins. Based on multilocus sequence analysis, the RSSC is categorized into four major lineages: Phylotype I (Asia), Phylotype II (Americas), Phylotype III (Africa) and Phylotype IV (Indonesia and surrounding regions). Recent comparative genomic studies have refined this classification into three distinct species: *R. pseudosolanacearum* (Lineages I and III), *R. solanacearum* (Lineage II) and *R. syzygii* (Lineage IV) (Prior et al. 2016). This lineage diversification is thought to result from geographic isolation and long‐term adaptation to diverse ecological niches. Lineage II, representing the ancestral group of RSSC, exhibits the greatest genetic diversity, whereas Lineage I is the most recently evolved and rapidly expanding clade (Sharma et al. 2022), indicating that each lineage may have developed distinct ecological and pathogenic adaptation strategies.

#### 2.1.2 Horizontal Gene Transfer and Genomic Plasticity

The genome of *R. solanacearum* exhibits remarkable plasticity, largely attributable to its distinctive bipartite structure, comprising a relatively conserved chromosome and a highly variable megaplasmid (Salanoubat et al. 2002). The megaplasmid is enriched in genes associated with environmental adaptability and pathogenicity and serves as a hotspot for genomic variation. Horizontal gene transfer (HGT) is a key evolutionary force driving the diversification of *R. solanacearum* pathogenicity. Through natural transformation, conjugation and phage‐mediated transduction, the RSSC could acquires exogenous DNA fragments efficiently (Guidot et al. 2009). Its genome harbours numerous genomic islands, prophages and integrative conjugative elements, which act as primary vehicles for HGT. These mobile genetic elements carry a variety of ‘ready‐to‐deploy’ functional modules, most notably type III effectors (T3Es), facilitating the rapid acquisition of novel virulence factors, antibiotic resistance genes and metabolic pathways. This genomic fluidity enables *R. solanacearum* to swiftly adapt to new hosts and environmental conditions.

#### 2.1.3 Dynamic Evolution of the Type III Effector Repertoire

Type III effectors (T3Es) are central virulence determinants employed by *R. solanacearum* to manipulate host cellular processes, with their diversity and functionality directly influencing host range and pathogenicity. Typically, *R. solanacearum* encodes an extensive repertoire of 60‐75 T3E genes, far exceeding the number identified in *Pseudomonas* or *Xanthomonas* species (Peeters et al. 2013). The effector repertoire follows a ‘core + variable’ model: approximately 30 core effectors are conserved across all strains, while the remainder are distributed among strains and lineages, contributing to host‐specific adaptations.

The T3E arsenal is evolutionarily dynamic, shaped by frequent gene acquisition, loss, duplication and functional diversification. For instance, the GALA family of effectors has undergone extensive duplication and sequence divergence within the RSSC, correlating with specialization to particular host plants (Remigi et al. 2011). Some effectors, such as AvrA and PopP1, are recognized by host resistance (R) proteins and act as avirulence (Avr) factors, thereby restricting the pathogen's host range. To overcome host immunity, *R. solanacearum* may evade recognition by mutating or deleting these Avr genes, enabling infection of previously resistant hosts (Poueymiro et al. 2009). This dynamic creates a critical evolutionary trade‐off. While possessing a broad effector repertoire can potentially expand host range, carrying an effector recognized by a specific host becomes a liability, restricting infection and thus potentially narrowing its overall ecological fitness.

Experimental evolution studies further highlight how genetic changes beyond canonical T3Es can expand host range. In a multihost serial passage experiment on the originally non‐host common bean ( *Phaseolus vulgaris* ), Guidot et al. (2014) showed that repeated inoculations led to the emergence of *R. solanacearum* lineages capable of infecting bean. Whole‐genome sequencing revealed parallel mutation in *efpR*, a global transcriptional regulator controlling both virulence and metabolic functions. Functional characterization confirmed that *efpR* mutations reprogram bacterial metabolism and enhance in planta fitness on bean, as further elucidated by Perrier et al. (2016). These findings indicate that host adaptation can arise through regulatory rewiring that indirectly modulates the expression or efficacy of the T3E arsenal, complementing the direct evolutionary turnover of effector genes. This rapid evolutionary ability directly poses a continuous challenge to breeding strategies that rely on a single resistance gene, highlighting the importance of understanding its evolutionary dynamics for the cultivation of long‐lasting resistance (as detailed in Section 3.1.1).

### 2.2 Environmental Survival Trade‐Offs: A Strategic Resource Manager

The *R. solanacearum* undergoes drastic environmental transitions during its life cycle, shifting from soil habitats to the internal vascular tissues of host plants. To thrive across these distinct ecological niches, the pathogen has evolved a complex regulatory network that facilitates adaptive trade‐offs. These regulatory systems enable *R. solanacearum* to allocate cellular resources efficiently, optimizing survival, proliferation and virulence in response to diverse and fluctuating environmental pressures.

#### 2.2.1 Quorum Sensing: Strategic Switching Between Growth and Pathogenic Modes

In RSSC, quorum sensing (QS) functions as a pivotal regulatory network for environmental adaptation. Central to this network is the Phc system, which responds to the population density‐dependent accumulation of the signalling molecule 3‐hydroxypalmitic acid methyl ester (3‐OH‐PAME). Upon reaching a threshold concentration, this signal activates the Phc system, which in turn coordinates the expression of over 100 downstream genes integral to virulence, metabolism and stress response (Schell 2000). This regulatory mechanism facilitates a biphasic lifestyle, switching between growth‐ and virulence‐oriented physiological states.

Complementing the cell‐density‐sensing Phc system, some lineages within the RSSC utilize a second, parallel QS system, Soll/R, which responds to N‐acyl homoserine lactone (AHL) signals (Li et al. 2024). This system is not universally conserved but is notably present in phylotype IIB strains, which include the important potato brown rot pathogen adapted to temperature climates. Specifically, the Soll/R quorum sensing system fine‐tunes the expression of key virulence‐associated genes, including *aidA*, *aidC* and *lecM*. This regulation is crucial for pathogenicity at lower temperatures; for instance, mutants in these genes show a significant loss of virulence at 20°C, an effect that is much less pronounced at the optimal temperature of 28°C (Meng et al. 2015). This mechanism is considered a key component of the ‘cool virulence’ strategy for these pathogens.

The activation of all such quorum sensing systems is governed by a fundamental principle: the perception of bacterial population density. This naturally divides the collective behaviour of the bacteria into two distinct phases. At low cell density (LCD), such as in the soil or during the initial stages of host colonization, the extracellular concentration of 3‐OH‐PAME remains below the activation threshold. Consequently, the global regulator PhcA is inactive. Under these conditions, the bacterium adopts a physiological state optimized for proliferation and dispersal, characterized by: (1) Enhanced metabolic versatility: The expression of genes involved in nutrient acquisition and catabolism is upregulated, facilitating the utilization of a diverse array of carbon and nitrogen sources (e.g., inositol, amino acids, organic acids). This broad metabolic capacity supports rapid growth and colonization in varied environments (Khokhani et al. 2017; Hamilton et al. 2021). (2) Increased motility and chemotaxis: Genes associated with flagellar synthesis and chemotaxis are highly expressed, enhancing the bacterium's ability to locate and move towards host roots (Tans‐Kersten et al. 2001). (3) Suppression of virulence factor expression: The production of metabolically expensive ‘public goods’, such as extracellular polysaccharides (EPS) and secreted degradative enzymes, is repressed. This conserves cellular resources, prioritizing them for biomass accumulation. Conversely, when the bacterial population proliferates to high cell density (HCD) within the confined host xylem, the 3‐OH‐PAME concentration exceeds the threshold and activates PhcA. This activation prompts a significant shift towards a pathogenic state, which involves a critical regulatory cascade: PhcA not only induces virulence factors but also actively represses PhcX, effectively shutting down the early colonization program (Liu et al. 2023). The PhcA‐on/PhcX‐off state includes: (1) Metabolic reprogramming: PhcA actively represses many metabolic pathways operative at LCD. Metabolic flux is redirected towards the catabolism of limited set of sugars, primarily sucrose and trehalose, which are abundant in the xylem sap (Peyraud et al. 2016; Hamilton et al. 2021). (2) Induction of key virulence factors: PhcA potently induces the expression of major virulence determinants, including EPS, plant cell wall‐degrading enzymes (PCWDEs) and the Type III secretion (T3SS). The coordinated expression of these factors is essential for systemic infection and symptom development (Genin and Denny 2012). (3) Downregulation of motility: Concurrently, flagellar gene expression and motility are repressed, promoting a transition from a planktonic state to a sessile, biofilm‐like lifestyle within the host vascular.

This QS‐mediated regulatory dichotomy represents a classic ecological trade‐off between proliferation and pathogenicity. At low cell density, the bacterium adopts a motile, metabolically versatile ‘explorer’ state, which maximizes its ability to find a host and colonize new niches, but at the cost of being non‐virulent. Conversely, activating the ‘pathogenic’ state at high density maximizes host exploitation and damage, but this commitment comes with the trade‐off of reduced motility and metabolic flexibility, making the population more vulnerable if the local host environment deteriorates. The ability to transition between a metabolic state favouring growth and a pathogenic state dedicated to host exploitation represents a critical component of its successful infection strategy and highlights its adaptive prowess as a major plant pathogen.

#### 2.2.2 Stage‐Specific Nutrient Utilization: Eating the Right Food at the Right Time

A prime example of adaptive resource allocation is the pathogen's ability to switch its metabolic priorities based on its location within the host, a strategy best described as ‘eating the right food at the right time’. This metabolic flexibility is crucial for navigating the distinct nutritional landscapes encountered during its life cycle. During the early stage, low‐density colonization of roots, the pathogen is metabolically tuned to utilize specific compounds secreted by the host root, such as inositol, which are crucial for its initial establishment (Hamilton et al. 2021). However, once it successfully invades the xylem and proliferates to high densities, a major metabolic reprogramming occurs. The bacterium then shifts its focus to catabolize the abundant resources within the xylem sap, primarily host‐derived sucrose and key amino acids. This switch is not merely for growth; the utilization of these specific nutrients is directly coupled to the massive production of virulence factors like EPS, which fuels the aggressive colonization and symptom development (Shen et al. 2020; Lowe‐Power et al. 2018). This strategic metabolic shift ensures that cellular resources are optimally allocated at each stage of infection, maximizing both survival and virulence. The precise molecular evidence underpinning this switch, as revealed by powerful multi‐omics approaches, provides a deeper understanding of its regulatory logic and will be discussed further in Section 2.2.4.

#### 2.2.3 Diversified Respiratory Strategies: Adapting to the Xylem Microenvironment

The plant xylem is not a uniform environment but rather a heterogeneous microhabitat characterized by steep oxygen gradients. The rapid proliferation of *R. solanacearum* within this confined space leads to localized oxygen depletion, generating hypoxic or even anoxic niches (Dalsing et al. 2015). To sustain energy production under these conditions, *R. solanacearum* has evolved the capacity for denitrification respiration, utilizing it as an alternative terminal electron acceptor. Recent findings by Truchon et al. (2023) revealed that distinct lineages of *R. solanacearum* have evolved divergent respiratory strategies. Strains from phylotypes I and III possess complete denitrification pathways, enabling the reduction of nitrate (NO_3_^−^) to dinitrogen gas (N_2_), a capability essential for full virulence. These complete denitrifiers also exhibit enhanced biofilm formation and a pronounced preference for hypoxic environments, suggesting superior adaptation to oxygen‐depleted niches, such as the interior of biofilms. In contrast, strains from phylotypes II and IV naturally lack the nosZ gene required for the final step of denitrification and thus can only reduce nitrate to nitrous oxide (N_2_O). Despite this limitation, these strains maintain high levels of virulence, implying that they may rely on alternative strategies, such as exploiting residual oxygen or occupying microenvironments within the xylem that retain relatively higher oxygen levels. This diversification of respiratory metabolism underscores fine‐scale niche partitioning and adaptive radiation within the *R. solanacearum* species complex in response to spatial heterogeneity within a shared host environment. This precise switching between the “growth” and “attack” modes mediated by the QS system is not only the key to its success but also reveals a regulatory centre that can be precisely targeted, providing a theoretical basis for anti‐toxicity strategies (such as population extinction) (as detailed in Sections 3.2.2).

#### 2.2.4 Multi‐Omics Reveal the Molecular Blueprint of Environmental Adaptation

Recent advances in multi‐omics technologies have revolutionized our ability to connect environmental stimuli directly to the pathogen's regulatory and metabolic responses with unprecedented resolution. These approaches provide a molecular blueprint that explains how *R. solanacearum* executes its adaptive strategies. For example, metabolomic and flux‐balance analyses have precisely mapped the pathogen's metabolic shifts, revealing its reliance on inositol during early root colonization versus sucrose and L‐glutamic acid within the nutrient‐rich xylem (Shen et al. 2020). This demonstrates a clear link between the local nutrient environment (stimulus) and metabolic reprogramming for virulence (response). Crucially, transcriptomics (RNA‐seq) provides a dynamic view of this adaptation. Studies have shown that upon exposure to specific environmental cues, such as changes in pH, oxygen levels, or the perception of plant‐derived signals, *R. solanacearum* undergoes massive transcriptional reprogramming (Jacobs et al. 2012; Zuluaga et al. 2013). For instance, transcriptomic analyses of *R. solanacearum* during infection have shown that, within the low‐oxygen host xylem environment, genes involved in denitrification and anaerobic respiration are significantly upregulated, reflecting a direct transcriptional adaptation to hypoxic stress. Mutants defective in key denitrification pathways exhibit reduced in planta growth and virulence, indicating that denitrifying respiration is a crucial survival strategy under these conditions (Dalsing et al. 2015; Truchon et al. 2022). Similarly, other RNA‐seq studies have captured the precise moment the PhcA regulon is activated, showing a coordinated up‐regulation of hundreds of virulence genes (e.g., those for EPS production and T3SS) and the simultaneous down‐regulation of genes for motility and early‐stage colonization (Perrier et al. 2018; Truchon et al. 2022). Collectively, these multi‐omics studies are moving us beyond descriptive biology to a predictive understanding of pathogen behaviour. They not only validate the ecological trade‐offs discussed previously but also pinpoint the specific genes and pathways that serve as the control hubs for these decisions, offering a wealth of new targets for intervention.

### 2.3 Interactions With Environmental Microorganisms: Survival in a Complex Social Network

The *R. solanacearum* does not exist in isolation; rather, it constantly interacts with diverse microbial communities both in the soil and within plant tissues. These interspecies interactions play a crucial role in shaping its survival, colonization efficiency and pathogenicity.

#### 2.3.1 Interaction With Fungi: Constructing a “Noah's Ark”

A pioneering study uncovered a novel mode of interaction between *R. solanacearum* and soil fungi (Spraker et al. 2016). The GMI1000 strain of *R. solanacearum* produces a unique lipopeptide secondary metabolite, ralsolamycin, which acts as a signalling molecule that induces the formation of chlamydospores, thick‐walled, stress‐resistant dormant structures, in several soil fungi, including *Aspergillus* and *Fusarium* species. Remarkably, *R. solanacearum* can subsequently invade and colonize these induced chlamydospores.

This ‘induce‐and‐invade’ strategy has profound ecological implications. The robust chlamydospore wells act as protective barriers, shielding the intracellular bacteria from environmental stresses such as desiccation, ultraviolet radiation, microbial predation and chemical exposure. This interaction may partly account for the bacterium's remarkable capacity to persist in soil over prolonged periods in the absence of host plants. The ability to co‐opt other microorganisms as protective niches represents a striking example of the pathogen's sophisticated environmental adaptability.

#### 2.3.2 Competition and Cooperation With Other Bacterial Society

In the densely populated rhizosphere and soil microenvironment, *R. solanacearum* is subjected to a range of powerful antagonistic pressures. On one hand, it engages in direct inter‐bacterial warfare for limited nutrients and spatial niches. To this end, it is equipped with a diverse arsenal of competitive strategies, such as the Type VI secretion system (T6SS) which functions as a molecular “toxic spear”, injecting effector proteins directly into neighbouring bacterial cells to eliminate competitors (Bernal et al. 2018). On the other hand, it faces a profound evolutionary challenge from viral predators (bacteriophages), which forces a trade‐off between survival and pathogenicity. Furthermore, it faces a profound evolutionary trade‐off when confronted by viral predators (bacteriophages). As demonstrated by Wang et al. (2024), gaining resistance to phage infection often requires mutations in Type IV pili. This adaption ensures survival against predation, but it comes at a high cost: since these pili are also essential virulence factors, the phage‐resistant survivors become significantly less virulent. This resistance‐virulence trade‐off is a powerful example of the ecological constraints shaping the pathogen's evolution and offers a potential avenue for biocontrol strategies.

In stark contrast to these antagonistic interactions, *R. solanacearum* can forge synergistic alliances that transform the microbial landscape in its favour, providing a clear mechanism for the formation of ‘disease‐conducive soils’. A compelling case is the interaction with so‐called ‘pathogen helpers’. Groundbreaking research from Li et al. (2022) demonstrated that *Phyllobacterium ifriqiyense* LM1 and *Microbacterium paraoxydans* LM2 were systematically identified as representative pathogen helpers of *R. solanacearum* . In vitro assays showed that these two strains increased pathogen proliferation by approximately 51% and 40%, respectively. In tomato rhizosphere co‐cultivation experiments, LM1 and LM2 elevated pathogen population densities by near 946% and 462%, respectively, and significantly exacerbated disease severity in the host plant (Pi: +75%; Mp: +62.5%) compared with the control treatment. Further interaction experiments revealed that suppression of pathogen helpers by other rhizosphere microbes effectively reduced pathogen abundance indirectly, underscoring that disease management strategies should extend beyond directly targeting the pathogen itself to include manipulation of helper taxa within the rhizosphere microbial community. This helper‐pathogen dynamic, which mechanistically underpins the formation of “disease‐conducive soils”, stands in stark contrast to the well‐documented phenomenon of ‘Suppressive soils’ (Mendes et al. 2011). This suppression is not a passive process but an active microbial warfare. Key players, often from the genera *Pseudomonas*, *Bacillus* and *Streptomyces*, deploy a range of strategies. These include the production of potent antibiotics and volatile organic compounds (VOCs) that directly inhibit *R. solanacearum* growth (Zhao et al. 2023; Tahir et al. 2017). Furthermore, these beneficial microbes engage in intense competition for nutrients and space. For instance, the production of high‐affinity siderophores by certain *Pseudomonas* strains can sequester iron, a critical element for the pathogen's proliferation, effectively starving it out (Gu et al. 2020; Shao et al. 2024). Beyond direct confrontation, many of these microbes act as ‘bodyguards’ for the plant, triggering Induced Systemic Resistance (ISR), which pre‐arms the plant's own immune system against subsequent pathogen attack (Pieterse et al. 2014). Therefore, the balance between pathogen helpers and suppressive microbes determines the ultimate trajectory of disease. Deciphering these complex interactions, from antagonism to facilitation, represents a promising avenue for the ecological control of bacterial wilt. This complex ‘social network’ determines the ‘disease‐suppressing’ or ‘disease‐promoting’ potential of the soil, revealing the great potential of indirectly controlling pathogenic bacteria through the management of microbial communities. This is the theoretical basis for ecological regulation and the development of microbial agents.

## 3 Re‐Evaluating Management Strategies

**FIGURE 3:**
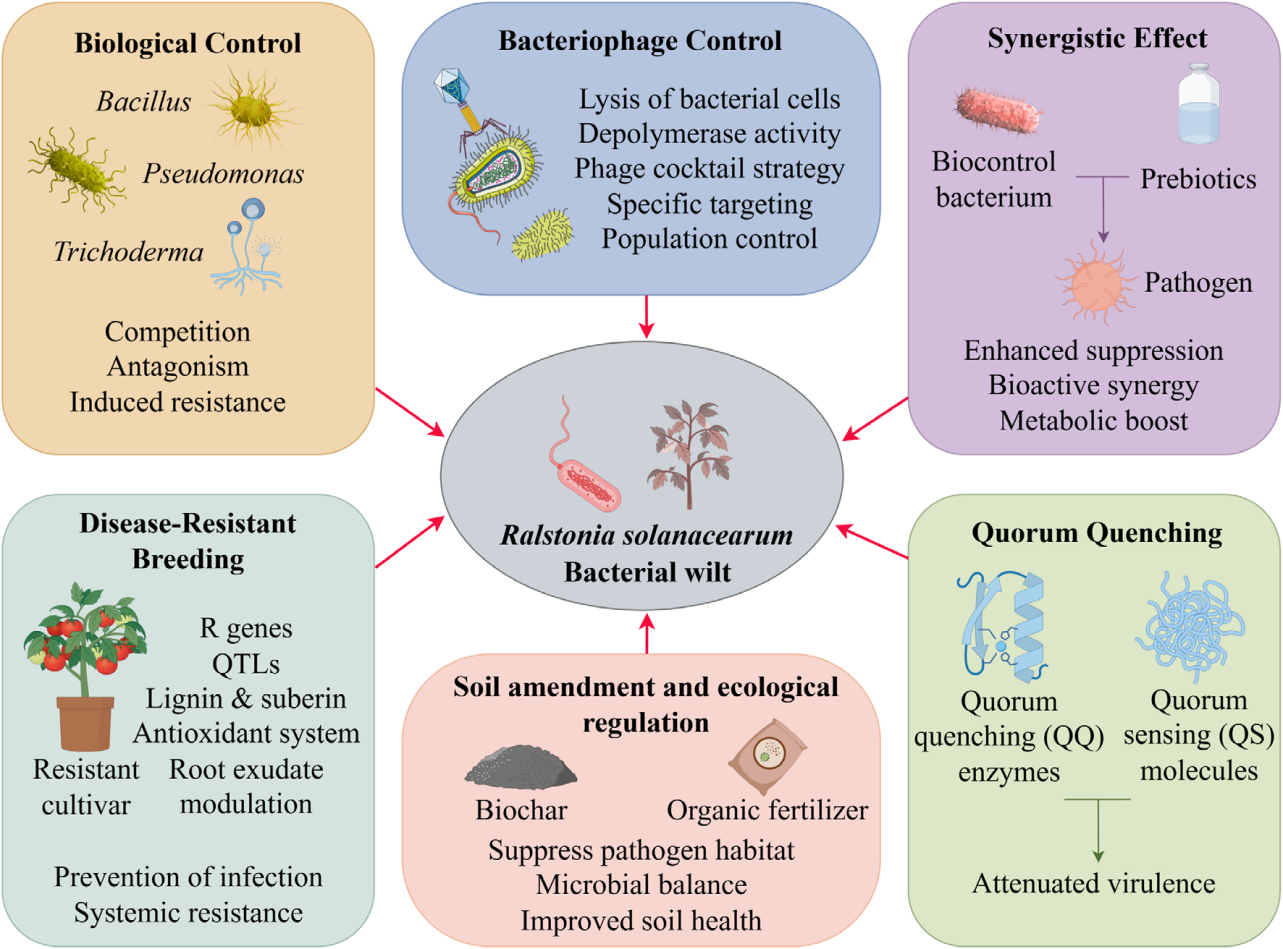
Integrated mechanisms for the control of *Ralstonia solanacearum* .

Due to the remarkable adaptability and diverse survival strategies of *R. solanacearum* , single control measures often fail to provide satisfactory and durable disease suppression. As a result, the integrated application of multiple complementary approaches, collectively known as Integrated Disease Management (IDM), is now widely regarded as the most effective strategy for controlling bacterial wilt (Figure 3). The following section provides a systematic overview and critical evaluation of major categories of existing control measures (Table 1).

**TABLE 1:** Summary of control approaches for management of *Ralstonia solanacearum* .

Control strategy	Specific method/agent	Targeted adaptive trait of pathogen	Ecological rationale	References
Biological control	The volatile organic compounds (VOCs) produced by *Bacillus amyloliquefaciens* SQR‐9 or *Bacillus velezensis* EM‐1	Physiological Adaptation (Quorum Sensing)	The VOCs function as Quorum Quenching (QQ) agents. They interfere with the PhcA‐mediated QS system, effectively ‘disarming’ the pathogen population. This prevents the coordinated switch from a low‐density (motile/proliferative) state to high‐density (virulent/EPS‐producing) state, thus blocking pathogenesis at a regulatory enzymes.	(Raza et al. 2016; Sui et al. 2022)
	*Pseudomonas putida* A1; *Pseudomonas fluorescens*	Ecological Adaptation (Interspecific Competition)	Employs a multi‐pronged attack on the pathogen's niche. (1) Niche Exclusion: Outcompetes the pathogen for space and nutrients on the root surface. (2) Resource Depletion: Secretes high‐affinity siderophores to sequester iron, starving the pathogen. (3) Host Modulation: Triggers Induced Systemic Resistance (ISR), turning the host plant into a less hospitable environment	(Sun et al. 2017; Abd El‐Wahed et al. 2023)
Bacteriophage control	Lytic bacteriophage, PQ43W; The ‘Phage Cocktail’ Strategy Lytic phages, RpT1 and RpY2; RsPod1EGY Phage	Evolutionary Adaptation (Rapid Evolution and Genomic Plasticity)	Imposes strong, multi‐directional predation pressure. This strategy directly counters the pathogen's ability to quickly evolve resistance to a single phage. It exploits the resistance‐virulence trade‐off, as mutations conferring resistance to one phage may compromise fitness or virulence, while evading multiple phages simultaneously is evolutionarily difficult.	(Huang et al. 2024; Wang et al. 2019; Thapa Magar et al. 2022; Elhalag et al. 2025)
Soil amendment and ecological regulation	Biochar: wheat straw, cassava husk, bean straw, sugar cane straw and corn straw	Ecological Adaptation (niche establishment and persistence in the soil environment)	This strategy manipulates the soil environment to make it inhospitable for pathogen and favourite for its antagonists. It works on two levels: (1) Biotic Suppression: Alters the soil's microbial landscape by promoting the growth of a diverse community of native beneficial microbes. This intensifies resource competition (for nutrients and space) and microbial antagonism (via antibiotics, VOCs, lytic enzymes), creating a ‘disease‐suppressive soil’ that actively limits the pathogen's survival and proliferation. (2) Abiotic Stress: Directly alters soil physicochemical properties (e.g., pH, nutrient availability, water retention) to create conditions that are suboptimal for *R. solanacearum* , thereby imposing physiological stress and reducing its fitness before it can even reach the host.	(Shuang et al. 2021; de Medeiros et al. 2022; Gu et al. 2017)
Disease‐Resistant Breeding	Screening and selection of elite genotypes; Gene editing technology; Whole Genome Resequencing	Physiological Adaptation (the pathogen's molecular machinery for host invasion and manipulation)	This strategy ‘hardens’ the host plant, turning it into a non‐permissive environment by enhancing its innate immune system. The rationale is to directly counter the pathogen's infection tools: (1) Effector‐Triggered Immunity (ETI): The primary mechanism. Resistance (R) proteins are bred into the plant to recognize specific virulence effector proteins secreted by the pathogen. This recognition triggers a rapid and powerful defence cascade, often including the Hypersensitive Response (HR), which leads to localized cell death to trap and kill the pathogen at the site of invasion. (2) Pattern‐Triggered Immunity (PTI): Breeding can also enhance the plant's ability to recognize general bacterial molecules (PAMPs), leading to a stronger and faster basal defence response (e.g., cell wall fortification, production of antimicrobial compounds), effectively stopping the pathogen before it can establish a significant infection.	(Rane et al. 2024; Wu et al. 2024; Zhang et al. 2023)
Quorum Quenching	*Bacillus toyonensis* AA1EC1 disrupts Quorum sensing in *R. solanacearum*	Physiological Adaptation (Quorum Sensing, the cell‐density dependent regulatory system)	This is a ‘signal jamming’ or ‘disarmament’ strategy that targets the pathogen's communication system. Instead of killing the bacteria, it prevents them from launching a coordinated attack. (1) Signal Interference: QQ agents either degrade the pathogen's signalling molecules or block their receptors. (2) Arresting the Virulence Switch: This interference tricks the bacterial population into perceiving itself as being at a low cell density. The pathogen fails to upregulate key virulence factors like EPS, cellulases and biofilm components, rendering the population collectively avirulent even at high numbers.	(Roca et al. 2024)

### 3.1 Countering Evolutionary Adaptation: The Host‐Pathogen Arms Race

#### 3.1.1 Resistance Breeding

The development of resistant cultivars represents a direct countermeasure to the pathogen's evolutionary adaptation, specifically targeting its arsenal of virulence factors like Type III effectors. However, the success of this strategy is constantly challenged by the dynamic evolution of the effector repertoire (as discussed in Section 2.1.3), which can lead to the breakdown of resistance. Therefore, modern breeding is shifting from simple screening to strategies that anticipate and manage the pathogen's evolutionary plasticity. The development and deployment of resistant cultivars remain the most cost‐effective, efficient and environmentally sustainable strategy for managing bacterial wilt. Resistance to *R. solanacearum* has been identified in several cultivated crops and their wild relatives, including Hawaii 7996 in tomato (Xu et al. 2023), *Solanum phureja* in potato (Lopes et al. 2021) and ‘EG203’ in eggplant (Nurdika et al. 2023). These genetic resources serve as the foundation for resistance breeding programs. However, many of these resistance resources are constrained by limitations such as low or partial resistance levels, instability under field conditions, and undesirable linkage with inferior agromonic traits, posing challenges for their direct use in commercial cultivars (Huet 2014). Resistance to bacterial wilt is generally quantitative, governed by multiple genes or quantitative trait loci (QTLs). Numerous QTLs associated with resistance have been identified and mapped in crops such as tomato, tobacco and eggplant (Wang et al. 2013; Lebeau et al. 2013). While quantitative resistance tends to confer greater durability, its incorporation into elite cultivars poses significant breeding challenges. In contrast, a few instances of single‐gene‐mediated qualitative resistance have been reported, exemplified by the *RRS1‐R* gene in *Arabidopsis thaliana* (Deslandes et al. 2002).

#### 3.1.2 Genetic Engineering

The resistance observed in certain cultivars results from a combination of physical and biochemical defence mechanisms. Structurally, resistant plants rapidly induce the formation of tyloses, gels and callose deposits within xylem vessels infection, effectively obstructing the systemic spread of *R. solanacearum* (Shi et al. 2023). At the cell wall level, resistant varieties often exhibit enhanced mechanical barriers and can more efficiently induce lignification and suberization in response to pathogen attack (Kashyap et al. 2022). Chemically, these plants frequently accumulate antimicrobial secondary metabolites, such as phenolics, flavonoids and alkaloids, that exert direct inhibitory effects on pathogen growth (Zhao et al. 2011). Besides, marker‐assisted selection (MAS) has greatly accelerated the pyramiding of multiple resistance QTLs into elite cultivars. Effector‐assisted breeding represents an emerging precision strategy that leverages pathogen effector repertoires as molecular probes to screen plant germplasm capable of recognizing specific effectors, thereby facilitating the discovery of broad‐spectrum and high‐efficiency resistance sources (Huet 2014). In parallel, gene editing technologies such as CRISPR/Cas9 offer unprecedented precision for modifying susceptibility (S) genes or enhancing the expression of resistance genes, opening new avenues for the development of durable resistance to *R. solanacearum* .

### 3.2 Disrupting Physiological Adaptation: Targeting Pathogen Regulation and Metabolism

#### 3.2.1 Modern Chemical Control

The limited and often inconsistent performance of chemical control against *R. solanacearum* can be traced to the pathogen's diverse strategies, including physical protection within plant vascular tissues and soil microhabitats, as well as resistance arising from genetic and regulatory plasticity. These features reduce pathogen exposure to lethal chemical concentrations and undermine the long‐term effectiveness of conventional bactericidal approaches. Consequently, contemporary chemical strategies are increasingly shifting from directly killing the pathogen to targeting its adaptive regulatory networks, for example by using quorum‐sensing inhibitors (QSIs) to disarm virulence rather than eliminate cells outright.

Traditional chemical practices include soil drenching or foliar spraying with copper‐based bactericides (e.g., copper hydroxide, copper oxychloride) and antibiotics (e.g., streptomycin, oxytetracycline). Nevertheless, because *R. solanacearum* resides deep within soil matrices and plant vascular tissues, these agents often fail to reach effective concentrations at the sites of infection. Furthermore, the prolonged and intensive use of such chemicals not only results in limited disease control efficacy but also contributes to the emergence of antimicrobial resistance and raises concern regarding environmental contamination and food safety (Wang et al. 2023). Rather than exerting direct bactericidal activity, certain chemical compounds function by activating the plant's innate immune system, particularly systemic acquired resistance (SAR), to enhance defence responses against invading pathogens (Gao et al. 2021). For example, salicylic acid (SA) analogues such as acibenzolar‐S‐methyl (ASM), as well as specific oligosaccharides and peptides, have been shown to confer resistance to bacterial wilt (Pradhanang et al. 2005). This indirect, host‐mediated strategy is generally considered more environmentally compatible and less likely to drive resistance evolution, representing a promising direction for next‐generation chemical disease management.

Recent advances in elucidating the pathogenic mechanisms of *R. solanacearum* have facilitated the development of targeted inhibitors against key virulence factors and regulatory systems. One emerging strategy involves quorum sensing inhibitors (QSIs), which disrupt the bacterium's 3‐hydroxypalmitic acid methyl ester (3‐OH‐PAME) signalling pathway (Lu et al. 2022). By interfering with this quorum sensing system, QSIs prevent the transition from a saprophytic to a pathogenic lifestyle, effectively ‘disarming’ the pathogen without exerting direct bactericide effects. Various natural compounds (e.g., coumarin, caffeic acid) and synthetic molecules have demonstrated QSI activity, significantly attenuating virulence (Qais et al. 2021; Li et al. 2021). This anti‐virulence approach represents a sustainable and resistance‐mitigating alternative to conventional chemical control.

#### 3.2.2 QS Inhibitors

Quorum quenching (QQ) represents one of the most illustrative examples of strategies that target pathogen environmental adaptation mechanisms. Rather than killing bacterial cells directly, this approach interferes with quorum‐sensing (QS) signalling (see Section 2.2.1), thereby preventing the coordinated transition of the pathogen population from a survival‐oriented state to a virulence expressing state and achieving effective disease suppression. *Ralstonia solanacearum* initiates infection by first releasing chemical signalling molecules, such as acyl‐homoserine lactones (AHLs), to communicate with neighbouring cells. When the local population density reaches a threshold concentration, referred to as a ‘quorum’, the bacteria collectively activate virulence genes and launch an attack, a process known as quorum sensing (QS). Once this ‘arsenal’ is triggered, the pathogen produces large quantities of extracellular polysaccharides, forms biofilms, secretes degradative enzymes and synthesizes phytotoxins, all of which lead to rapid plant wilting and death. QQ refers to the disruption of QS signalling, and has emerged as a key strategy to disarm *R. solanacearum*. A recent study demonstrated that *Bacillus toyonensis* AAEC1 can continuously secrete quorum‐quenching enzymes, most notably AHL‐lactonase. This enzyme specifically recognizes and cleaves the conserved lactone ring structure of AHL molecules, thereby abolishing their signalling function (Roca et al. 2024). As a result, although *R. solanacearum* cells may remain in the rhizosphere, they lose the ability to coordinate virulence expression. Compared to conventional antibiotic treatments, this anti‐virulence strategy imposes minimal selective pressure on pathogens, making it less likely to drive the emergence of resistance. It thus represents a smarter and more sustainable approach to bacterial wilt management.

### 3.3 Manipulating Ecological Adaptation: Managing the Agro‐Ecosystem

#### 3.3.1 Biocontrol and Microbiome Engineering

Microbial biocontrol strategies fundamentally operate by disrupting the pathogen's ecological adaptation (see Section 2.3). Whether through the introduction of antagonistic microorganisms that impose resource competition and direct antagonism, or through the application of bacteriophages that exert predation pressure, the shared objective is to disrupt pathogen‐favourable equilibria within the microscale ecosystem. Many rhizosphere bacteria, particularly strains belonging to the genera *Bacillus* and *Pseudomonas*, are among the most extensively studied and widely applied biocontrol agents against *R. solanacearum* (Sun et al. 2022). Their mechanisms of action are diverse and include: (1) antibiosis, involving the production of various antibiotics, lipopeptides (e.g., surfactin, iturin, fengycin) and volatile organic compounds (VOCs) that directly inhibit or kill *R. solanacearum* (Chen et al. 2020); (2) Competition for colonization sites on root surfaces and essential nutrients such as iron, carbon and nitrogen, often mediated by siderophore production (Abd El‐Wahed et al. 2023); (3) induced systemic resistance (ISR), whereby plant immune responses are triggered through bacterial surface components (e.g., flagellin, lipopolysaccharides) or secreted molecules, thereby enhancing host defence against subsequent pathogen attack (Fu et al. 2020). In addition to bacterial agents, members of the genus *Trichoderma* and certain arbuscular mycorrhizal fungi (AMF) also demonstrate strong biocontrol potential against *R. solanacearum* . *Trichoderma* species secrete extracellular enzymes such as chitinases and glucanases that degrade pathogen cell walls, along with a range of antimicrobial metabolites. Meanwhile, AFM enhance plant nutrient uptake and stress tolerance, and may confer protection against *R. solanacearum* through physical barrier formation and the induction of systemic plant resistance (Tahat et al. 2012).

Bacteriophages are viruses that specifically infect bacteria and exhibit high host specificity. The application of lytic phages targeting *R. solanacearum* has emerged as a promising biocontrol strategy. Phages can be applied directly to soil, irrigation water, or seedlings to suppress pathogen populations. However, under the selective pressure imposed by single‐phage treatments, bacteria can rapidly evolve resistance, resulting in diminished efficacy as resistant variants become dominant. Moreover, *R. solanacearum* populations in field soils are genetically diverse and structurally complex rather than uniform, further constraining the effectiveness of single‐phage approaches. To overcome these challenges, the use of ‘phage cocktails’ composed of multiple phage types has been proposed and widely adopted, enhancing the durability and stability of control efficacy (Ramírez et al. 2020; Thapa Magar et al. 2022).

#### 3.3.2 Agronomic Practices

Agronomic practices can be viewed fundamentally as a form of niche management, aimed at creating soil and cropping environments that are incompatible with the ecological adaptation of *R. solanacearum* . By manipulating host availability, resource distribution and soil microhabitats, these practices suppress pathogen survival and dissemination while promoting healthy crop development. For example, crop rotation directly disrupts host continuity, whereas soil amendments seek to reconfigure the soil microenvironment in ways that favour antagonistic and beneficial microorganisms. A four‐year study on tobacco‐maize (non‐host, Gramineae) rotation showed that rotation plots remained free of bacterial wilt symptoms, accompanied by increased microbial diversity and enhanced network stability (Ma et al. 2024). This practice disrupts the host‐pathogen cycle and can reshape the soil microbial community toward a more suppressive profile. In addition, intercropping with specific plants, such as marigold (*Tagetes* spp.), scallion ( *Allium fistulosum* ), or garlic, has been reported to suppress bacterial wilt (Hu et al. 2021; Vega et al. 2023), likely due to the antimicrobial compounds secreted by their roots. Grafting susceptible yet agronomically desirable scions onto resistant rootstocks is a widely adopted practice in vegetable production. For instance, grafting tomato plants onto bacterial wilt‐resistant rootstocks has been shown to effectively block root invasion by *R. solanacearum* while maintaining high fruit yield and quality (Louws et al. 2010).

Avoiding excessive flood irrigation and implementing water‐efficient systems such as drip irrigation can further limit pathogen dissemination via water movement. Proper nutrient management, particularly the application of calcium and silicon fertilizers, enhances cell wall rigidity and strengthens plant physical defences (Ali and Singh 2025). Additionally, adjusting soil pH through lime application to a neutral or slightly alkaline range (pH 6.5–7.5) creates unfavourable conditions for the acidophilic *R. solanacearum* , thereby contributing to disease suppression (Wang et al. 2023). Moreover, the application of well‐composted organic fertilizers or soil conditioners such as biochar enhances soil physicochemical properties and stimulates the activity of beneficial microorganisms, collectively fostering a disease‐suppressive soil microbiome that constrains pathogen establishment and proliferation.

#### 3.3.3 Physical Practices

Physical control strategies aim to directly eliminate or suppress soilborne pathogens by imposing extreme physical conditions that exceed their environmental tolerance thresholds, thereby directly targeting pathogen survival and persistence in soil. By modifying the physical properties of the soil environment, these approaches reduce primary inoculum sources and limit initial infection. Among the most straightforward and effective physical approaches, soil disinfection can be achieved through the application of high‐temperature steam, hot water or chemical fumigants (e.g., chloropicrin; methyl bromide was formerly used) (Gullino et al. 2022). These treatments effectively eliminate *R. solanacearum* along with a broad spectrum of soilborne pathogens, insect pests and weed seeds. As a more environmentally sustainable alternative, soil solarization involves covering moist soil with transparent polyethylene film during periods of solar radiation. This technique harnesses solar energy to elevate the temperature of the upper soil layers to pathogen‐lethal levels, thereby achieving effective disinfection (Hernández‐Lara et al. 2023). Soil solarization has demonstrated substantial efficacy in controlling bacterial wilt in tomato and other crops, particularly in regions with intense sunlight (Dai et al. 2020).

*R. solanacearum* can disseminate over long distances via contaminated propagative materials such as seed tubers and seedlings. Hence, the exclusive use of certified pathogen‐free planting materials is fundamental for preventing disease outbreaks and limiting the introduction of the pathogen into new areas. For vegetatively propagated crops such as ginger, hot water or hot air treatment of seed rhizomes at 50°C for 30 min has been shown to effectively eliminate latent infections (Tsang and Shintaku 1998). Additionally, biofumigation harnesses biologically active volatile compounds such as isothiocyanates, released during the decomposition of specific plant residues, particularly those from *Brassicaceae* species, to suppress soilborne pathogens. The incorporation of these plants into the soil as green manure has been shown to significantly reduce the survival of *R. solanacearum* (Khan et al. 2020). Physical control methods offer the advantage of broad‐spectrum and rapid efficacy. However, they often entail high operational costs (e.g., steam treatment) and may indiscriminately eliminate beneficial soil microorganisms, potentially disrupting microbial community balance. Furthermore, the effectiveness of soil solarization is highly dependent on environmental conditions, particularly temperature and solar radiation, which may limit its applicability in cooler or cloudier regions.

## 4 Challenges and Prospects

Despite significant progress in elucidating the adaptation mechanisms of *R. solanacearum* and in developing diverse control strategies, effective management of this persistent disease continues to face numerous formidable challenges, while simultaneously offering promising avenues for future research.

### 4.1 Challenges

The major challenges confronting current management strategies stem from the exceptional and multidimensional environmental adaptability of *R. solanacearum* . Genomic plasticity accelerates the erosion of host resistance, diverse survival strategies preclude complete eradication, and complex interactions with biotic and abiotic environments amplify the uncertainty of control outcomes.

The complex lineage and sequevar structure within the RSSC, combined with frequent gene recombination and horizontal gene transfer (HGT), drives continuous shifts in pathogenicity and host specificity. This high genetic variability poses a significant risk for the rapid breakdown of resistance conferred by single genes and undermines the long‐term efficacy of chemical control agents. Moreover, the absence of rapid and accurate typing methods for highly recombinant lineages further impedes effective disease surveillance and early‐warning systems (Sharma et al. 2022). In parallel, *R. solanacearum* can establish asymptomatic latent infections in resistant cultivars and weed hosts, functioning as cryptic inoculum reservoirs that are difficult to detect and manage. Its remarkable environmental persistence, mediated by biofilm formation in soil and water, as well as associations with fungi (e.g., survival within thick‐walled spores), renders complete eradication of field inoculum virtually impossible (Spraker et al. 2016). Disease incidence and severity are further shaped by interacting factors including host genotype, pathogen strain, soil properties, temperature and humidity. Elevated temperature and humidity notably exacerbate disease outbreaks and may compromise the resistance of certain cultivars. Under ongoing climate change, increased environmental variability is expected to further magnify uncertainty and management complexity. Although integrated disease management (IDM) is widely acknowledged as the most effective approach, optimizing combinations of resistant cultivars, agronomic practices, chemical inputs and biological control agents, both scientifically and economically, and translating them into protocols that are robust and readily adoptable by farmers remain formidable challenges. Notably, many biocontrol agents that perform well under controlled conditions show diminished and inconsistent efficacy in complex and heterogeneous field environments.

### 4.2 Future Research Directions

In response to the aforementioned challenges, future research should prioritize systematic, precise and forward‐looking approaches, with potential breakthroughs concentrated in the following key areas. Employ metagenomics and metatranscriptomics to comprehensively characterize the composition and functional dynamics of microbial communities in contrasting soil types (disease‐suppressive versus conducive), thereby identifying key taxa and interaction networks involved in bacterial wilt suppression. In parallel, integrate genomic epidemiology to establish a global surveillance system for the RSSC, enabling real‐time monitoring of the emergence and dissemination of novel virulence genes. Such efforts will provide critical data to inform the rational deployment of resistant cultivars and enhance early warning capabilities.

Advance beyond traditional QTL mapping and marker‐assisted selection (MAS) towards genome design breeding. Employ effector‐assisted breeding to identify and pyramid multiple resistance (R) genes with distinct recognition spectra, thereby constructing durable resistance gene pyramids. Leverage CRISPR/Cas gene‐editing technologies to precisely modify plant susceptibility (S) genes or optimize defence‐related pathways, such as cell wall biosynthesis and secondary metabolism, to develop novel resistant germplasms. Shift from screening individual biocontrol strains toward the design and assembly functionally stable synthetic microbial communities (SynComs). By combining strains with complementary traits, such as antibiotic production, induced systemic resistance, and nutrient competition, these ‘probiotic consortia’ can efficiently colonize the rhizosphere and provide consistent disease suppression, representing a transformative advancement in biological control. Deepen the molecular understanding of key biological processes in *R. solanacearum* , including quorum sensing (QS), biofilm formation and type III secretion system (T3SS). Develop highly specific, environmentally friendly inhibitors such as quorum sensing inhibitors (QSIs) and biofilm disruptors. Concurrently, actively explore nanotechnology applications for controlled pesticide release, targeted delivery and rapid pathogen detection (Wang et al. 2023). Integrate meteorological, soil physicochemical, crop growth modelling and pathogen surveillance data by leveraging artificial intelligence and big data analytics to develop predictive risk warning models for bacterial wilt. This approach will transform disease control from ‘reactive response’ to ‘proactive prevention’, enabling precision control of water, fertilizer, and pesticide applications, and ultimately achieving efficient, economical, and sustainable bacterial wilt management.

## 5 Conclusion

The RSSC, characterized by exceptional genetic diversity and environmental adaptability, remains a persistent threat to global agriculture, rendering any single ‘silver bullet’ control strategy unrealistic. Durable management of bacterial wilt will therefore depend on a new generation of integrated disease management (IDM) strategies explicitly informed by pathogen adaptation. Progress will require the development of more intelligent disease‐resistant crops through precision and evolution‐aware breeding, alongside the deliberate management of soil microbial communities via microbiome engineering. In parallel, advances in information technologies will be essential for accurate disease forecasting and targeted interventions. Ultimately, only by integrating mechanistic insights at the molecular level with ecosystem‐scale regulation and global surveillance frameworks can sustainably control of this highly adaptable ‘invisible enemy’ be achieved, thereby supporting global food security and resilient agricultural systems.

## Author Contributions

**Mingzhao Han:** writing – original draft, conceptualization. **Peng Li:** writing – review and editing, conceptualization, supervision. **Xin Liu:** visualization. **Guixiang Li:** writing – review and editing.

## Funding

This work was supported by The National Natural Science Foundation of China (No. 32260652).

## Conflicts of Interest

The authors declare no conflicts of interest.

## Data Availability

Data sharing not applicable to this article as no datasets were generated or analysed during the current study.
